# Neurological affection and serum neurofilament light chain in wild type transthyretin amyloidosis

**DOI:** 10.1038/s41598-024-60025-6

**Published:** 2024-05-02

**Authors:** Helena F. Pernice, Adrian L. Knorz, Paul J. Wetzel, Carolin Herrmann, Harisa Muratovic, Finn Rieber, Eleonora Asaad, Gunnar Fiß, Gina Barzen, Elisabeth Blüthner, Fabian Knebel, Sebastian Spethmann, Daniel Messroghli, Bettina Heidecker, Anna Brand, Christoph Wetz, Carsten Tschöpe, Katrin Hahn

**Affiliations:** 1 Charité – Universitätsmedizin Berlin, corporate member of Freie Universität Berlin and Humboldt-Universität zu Berlin, Amyloidosis Center Charité Berlin (ACCB), Charitéplatz 1, 10117 Berlin, Germany; 2 Charité – Universitätsmedizin Berlin, corporate member of Freie Universität Berlin and Humboldt-Universität zu Berlin, Department of Neurology and Experimental Neurology, Charitéplatz 1, 10117 Berlin, Germany; 3 Charité – Universitätsmedizin Berlin, corporate member of Freie Universität Berlin and Humboldt-Universität zu Berlin, Institute of Biometry and Clinical Epidemiology, Charitéplatz 1, 10117 Berlin, Germany; 4 Charité–Universitätsmedizin Berlin, Corporate Member of Freie Universität Berlin and Humboldt-Universität zu Berlin, Charitéplatz 1, 10117 Berlin, Germany; 5 Deutsches Herzzentrum der Charité, Department of Cardiology, Angiology and Intensive Care Medicine, Charitéplatz 1, 10117 Berlin, Germany; 6 DZHK (German Centre for Cardiovascular Research), Partner Site Berlin, Berlin, Germany; 7 Charité – Universitätsmedizin Berlin, corporate member of Freie Universität Berlin and Humboldt-Universität zu Berlin, Medical Clinic m.S. Hepatology and Gastroenterology CCM/CVK, Berlin, Germany; 8 Klinik für Innere Medizin mit Schwerpunkt Kardiologie, Sana Klinikum Lichtenberg, Berlin, Germany; 9 Deutsches Herzzentrum der Charité, Department of Cardiology, Angiology and Intensive Care Medicine, Augustenburger Platz 1, 13353 Berlin, Germany; 10 Deutsches Herzzentrum der Charité, Department of Cardiology, Angiology and Intensive Care Medicine, Hindenburgdamm 30, 12203 Berlin, Germany; 11 Charité – Universitätsmedizin Berlin, corporate member of Freie Universität Berlin and Humboldt-Universität zu Berlin, Department of Nuclear Medicine, Hindenburgdamm 30, 12203 Berlin, Germany; 12 Berlin Institute of Health at Charité (BIH)–Universitätsmedizin Berlin, Charitéplatz 1, 10117 Berlin, Germany

**Keywords:** Neurological disorders, Peripheral neuropathies, Heart failure, Biomarkers

## Abstract

In contrast to inherited transthyretin amyloidosis (A-ATTRv), neuropathy is not a classic leading symptom of wild type transthyretin amyloidosis (A-ATTRwt). However, neurological symptoms are increasingly relevant in A-ATTRwt as well. To better understand the role of neurological symptoms in A-ATTRwt, A-ATTRwt patients were prospectively characterized at Amyloidosis Center Charité Berlin (ACCB) between 2018 and 2023 using detailed neurological examination, quality of life questionnaires, and analysis of age- and BMI-adapted serum neurofilament light chain (NFL) levels. 16 out of 73 (21.9%) patients presented with a severe neuropathy which we defined by a Neuropathy Impairment Score (NIS) of 20 or more. In this group, quality of life was reduced, peripheral neuropathy was more severe, and spinal stenosis and joint replacements were frequent. Age- and BMI matched serum NFL levels were markedly elevated in patients with a NIS ≥ 20. We therefore conclude that highly abnormal values in neuropathy scores such as the NIS occur in A-ATTRwt, and have an important impact on quality of life. Both peripheral neuropathy and spinal canal stenosis are likely contributors. Serum NFL may serve as a biomarker for neurological affection in patients with A-ATTRwt. It will be important to consider neurological aspects of A-ATTRwt for diagnosis, clinical follow-up, and future treatment development.

## Introduction

Transthyretin amyloidosis (A-ATTR) is a systemic disease that mainly presents as cardiomyopathy and/or neuropathy. In contrast to inherited A-ATTR (ATTRv, v for variant), in which the phenotype can be purely or predominantly neurological, wild type A-ATTR (ATTRwt) is historically known as a late onset cardiomyopathy primarily affecting elderly men^1^. As we and others have reported previously, neurological symptoms in A-ATTRwt are generally considered mild and present as preliminarily sensory peripheral neuropathy^2–6^. The characteristics of neuropathy are usually described as a length-dependent, axonal sensory neuropathy, which only mildly affects the ability of ambulation^2,5^. Co-occurring small fiber neuropathy (SFN) has also been described in patients with A-ATTRwt^4,5,7^. However, with the rising attention to neurological aspects of A-ATTRwt, individual cases of atypically severe neuropathy and extracardiac amyloid deposition in tissues such as skeletal muscle have been reported^8–11^. In A-ATTRv, severity of neuropathy correlates with serum neurofilament light chain (sNFL) levels^12–14^. sNFL is a cytoskeletal scaffolding protein released by axons upon nerve injury and has been used as a promising biomarker in several neurological disorders^12,13^. At the Amyloidosis Center Charité Berlin (ACCB) registry study we perform neurological and cardiological screening visits on all patients independent of the cardinal symptom. Surprisingly, several patients showed atypically high Neuropathy Impairment Scores (NIS) of 20 points or more. To our knowledge, no study has systematically investigated the role of severe neurological affection in A-ATTRwt patients. The goal of this study was to characterize the prevalence, clinical presentation, and etiology of severele neurological symptoms in our ACCB register cohort. Due to the increasing relevance of sNFL as a biomarker for neurological affection, especially in A-ATTRv, and the lack of systematic literature on the role of sNFL in A-ATTRwt, we set a focus on investigating the relevance of sNFL in our cohort of patients with A-ATTRwt.

## Results

### Demographics and general characteristics

We prospectively included 73 patients that presented at ACCB between 2018 and 2023 into this study. 16 patients (21.9%) had an abnormally high clinical neuropathy score (NIS of 20 points or more). Demographics of the cohort are depicted in Table 1. The median age was 83.0 (IQR 6.0) years with a range of 68 to 91 years, and the cohort included 7 women (15.3%). Patients with NIS < 20 (median 83.0, interquartile range [IQR] 6.0) were recruited at similar age as patients with NIS ≥ 20 (median 82.0, IQR 6.0). Median onset of cardiac symptoms as reported by the patient was at the age of 75.0 (IQR 23.3) years overall, 78.0 (IQR 16.0) years for patients with NIS < 20, and 61.0 (IQR 20.0) years for patients with NIS ≥ 20). The median latency from first symptom to diagnosis of A-ATTRwt was 16.0 (IQR 28.0) months overall, and was similar between patients with NIS < 20 (15.0, IQR 28.0 months) and patients with NIS ≥ 20 (19.0, IQR 30.3 months).

**Table 1:** Demographics patient cohort.

	Entire cohort	n	NIS < 20	n	NIS ≥ 20	n
Number of patients, *n* (% of entire cohort)	73 (100)		57 (78.1)		16 (21.9)	
Sex female, *n* (% of subgroup)	7 (9.5)		5 (8.7)		2 (12.5)	
Median age, years (IQR)	83.0 (6.0)	73	83.0 (6.0)	57	82.0 (6.0)	16
Median age at symptom onset, years (IQR)	75.0 (23.3)	34	78.0 (16.0)	24	61.0 (20.0)	10
Median latency of diagnosis, months (IQR)	16.0 (28.0)	35	15.0 (28.0)	25	19.0 (30.0)	10
Median NYHA score (IQR)	2.0 (1.0)	59	2.0 (1.0)	45	2.0 (1.0)	14
Median NTproBNP, ng/L (IQR)	2351.0 (3193.0)	68	2330.0 (2280.0)	53	4117.0 (5879.0)	15
Median H/CL ratio (IQR)	2.5 (1.0)	34	2.6 (1.0)	22	2.3 (1.0)	12
Median HbA1c, % (IQR)	5.9 (0.8)	45	5.8 (0.7)	33	6.2 (1.2)	12

Higher NIS relate to more severe neurological affection. Separate n-number columns for each group indicate how many patient datasets were available.

Patients with NIS < 20 and NIS ≥ 20 had similar cardiac affection measured by New York Heart Association (NYHA) score, and cardiac uptake on Technetium-99 m-labelled 3,3-diphosphono-1,2-propanodicarboxylic acid (DPD)-scintigraphy measured by heart to contralateral ratios (see Table 1). While median values of N-terminal prohormone of brain natriuretic peptide (NTproBNP) suggested a more severe affection of patients with higher neurological affection (NIS < 20: 2330.0, IQR 2280.0 ng/L; NIS ≥ 20: 4117.0, IQR 5879.0 ng/L), there was an important spread in both groups causing this difference to be not statistically significant (p = 0.178). Also, glycated hemoglobin (HbA1c) values did not differ relevantly between the groups, and none of the patients had vitamin B12 deficiency.

### Quality of life in relation to neurological affection

**Figure 1:**
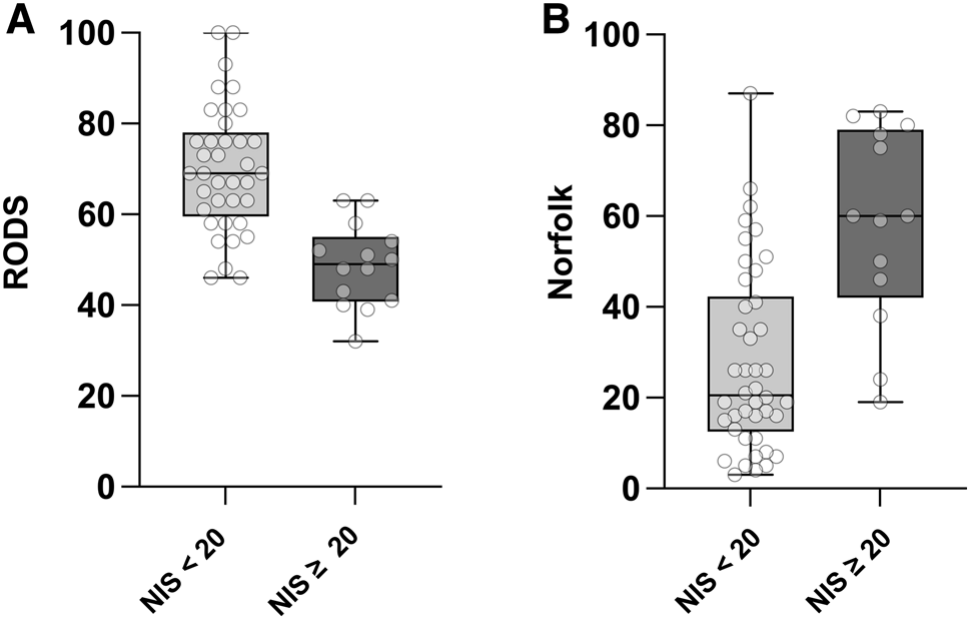
Quality of life analysis of patients with severe neuropathy. Boxplots of (**A**) RODS and (**B**) Norfolk questionnaires show reduced quality of life and self-sufficiency in patients with NIS ≥ 20 compared to patients with NIS < 20. Statistics: (**A**) Unpaired t-test, p < 0.0001; NIS < 20: n = 37, NIS ≥ 20: n = 14. (**B**) Mann–Whitney-U test, p < 0.0001; NIS < 20: n = 42, NIS ≥ 20: n = 13.

To evaluate whether the neurological affection measured by the NIS has an impact on quality of life and functional abilities, we compared results of the Rasch-Built-Overall-Disability Score (RODS) and Norfolk Quality of Life questionnaires across patients with NIS < 20 and NIS ≥ 20 (Fig. 1). Importantly, we were able to see prominent reductions of quality of life in both scores (p < 0.0001). The RODS, which focuses on the ability to perform daily life activities, showed worsening with increased severity of neuropathy (NIS < 20: mean 70.1, standard deviation [SD] 13.8, n = 37; NIS ≥ 20: mean 48.7, SD 9.1, n = 14; p < 0.0001, unpaired t-test, Fig. 1A). The Norfolk Quality of Life score, which captures more specific neurological symptoms with a focus on sensory symptoms, was more affected in patients with severe neuropathy and less in patients with mild neuropathy compared to NIS < 20 (NIS < 20: median 20.5, IQR 29.8, n = 42; NIS ≥ 20: median 60.0, IQR 37.0, n = 13; p < 0.0001, Mann–Whitney-Test, Fig. 1B).

### Neurological examination

**Figure 2:**
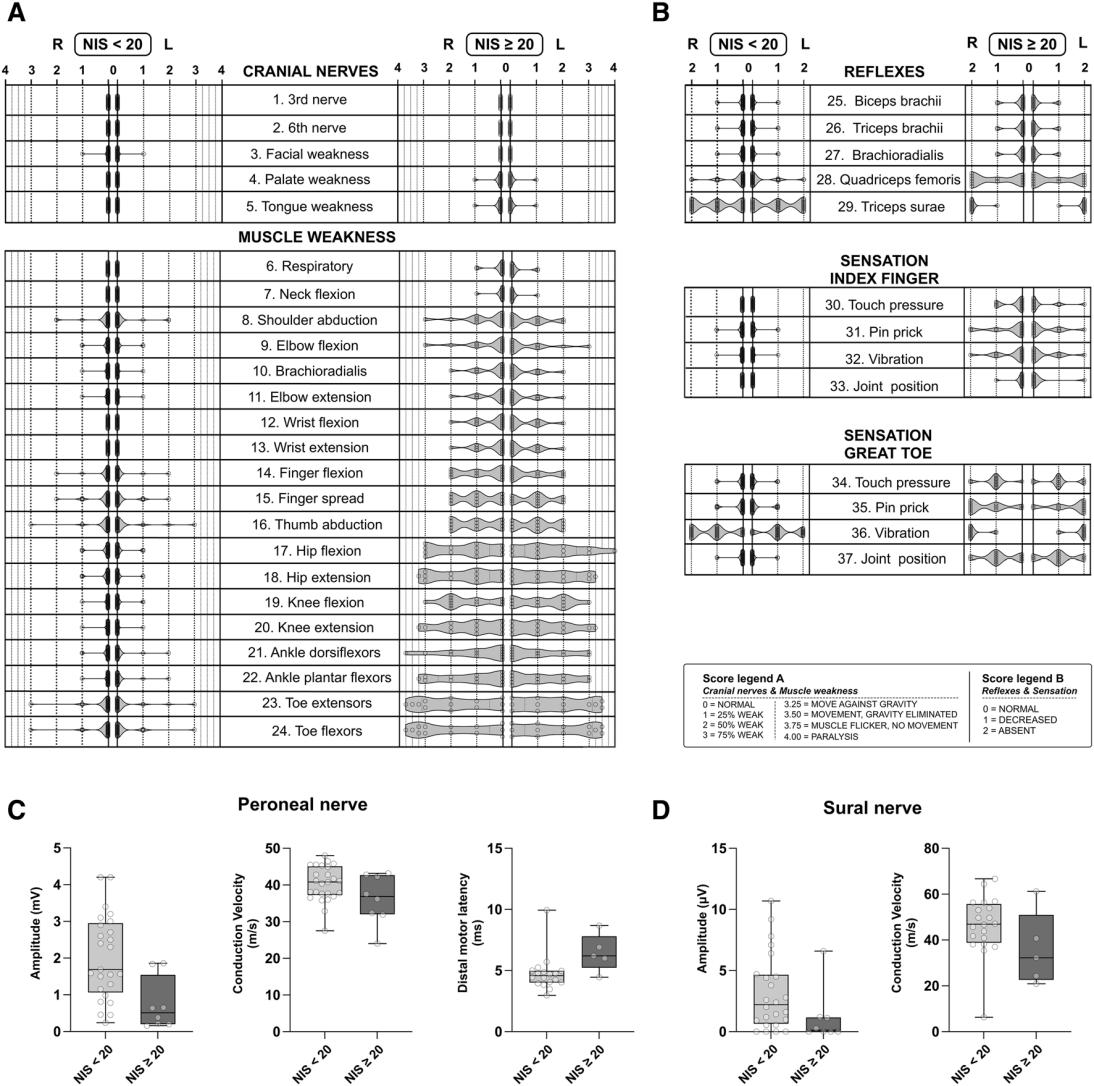
Neurological characterization of patients of different neurological severity. (**A**,**B**) Violin plots of NIS subitem scores. (**A**) Affection of cranial nerves and muscle weakness in patients with NIS < 20 vs NIS ≥ 20. Scores range from 0 to 4 (**B**) Affection of reflexes, and proximal and distal sensation in patients with NIS < 20 vs NIS ≥ 20. Scores range from 0–2. Data for both left (L) and right (R) body sides are displayed for all items. Detailed legends for both (**A**) and (**B**) are depicted in the Score legend A and Score legend B. NIS < 20: n = 33; NIS ≥ 20: n = 13. (**C**,**D**) Comparison of nerve conduction studies (NCS) between groups of different neurological affection (by NIS). Statistics: Mann–Whitney-U test. (**C**) Amplitude peroneal nerve: p = 0.035; conduction velocity peroneal nerve: p = 0.124; distal motor latency peroneal nerve: p = 0.015; NIS < 20: n = 25; NIS ≥ 20: n = 8. (**D**) Amplitude sural nerve: p = 0.034; conduction sural nerve: p = 0.109; NIS < 20: n = 24; NIS ≥ 20: n = 8.

Since quality of life strongly differed between the two groups (NIS < 20 and NIS ≥ 20), we next aimed at better understanding the clinical differences between the two groups. We therefore performed careful neurological examinations including detailed sensory and motor examinations on all patients. The NIS represents a sum score, which is derived from summation of its sub-items that measure cranial nerve function, motor strength, tendon reflex levels and distal sensation for light touch, vibration, pinprick and proprioception. To show in detail, which modalities are more affected in higher sum scores (NIS ≥ 20) versus lower sum scores (NIS < 20), we visualized the distribution of sub-items across the patient groups. Violin plots showing the mean score of each individual item of the NIS are depicted in Fig. 2A and B. Generally, patients showed a homogenous picture of a length-dependent neuropathy. In patients with higher scores, increased severity of distal sensory and motor deficits was observed, while the overall distribution of deficits remained similar. Specifically, control patients (NIS < 20) presented with mild sensory symptoms involving absent ankle reflexes and reduced vibration sensation of the distal lower limb (Fig. 2B), and mild paresis of the intrinsic finger muscles, and little limitation in proximal muscle strength (Fig. 2A). Patients with NIS ≥ 20 presented with more prominent decrease and often absence of sensation to vibration, pinprick, proprioception, and light touch in the lower extremities (Fig. 2B). Furthermore, there was pronounced lower and upper limb weakness frequently resulting in distal plegia with proximal paresis (Fig. 2A), and distal tendon reflexes were generally reduced or absent in the distal lower extremity. Cranial nerves and neck or respiratory muscles were only mildly affected in both groups.

In addition to clinical examination, we performed nerve conduction studies (NCS). Since clinical examination revealed most relevant deficits in the lower limbs, a comparison of lower limb NCS results between patients with NIS sum score of < 20 and patients with a NIS ≥ 20 is depicted in Fig. 2C and D. NCS of median, ulnar, and tibial nerves are summarized in Supplementary Table 1. Amplitudes of peroneal nerve motor action potentials (CMAPs) were markedly lower in patients with NIS ≥ 20 compared to patients with NIS < 20 (NIS < 20: median 1.7 mV, IQR 1.9, n = 25; NIS ≥ 20: median 0.51 mV, IQR 1.3, n = 8; p = 0.004, Mann–Whitney-Test) (Fig. 2C). Similarly, sural sensory nerve amplitudes (SNAPs) were reduced in patients with NIS ≥ 20 (NIS < 20: median 2.2 µV, IQR 4.0, n = 24; NIS ≥ 20: median 0.1 µV, IQR 1.2, n = 8; p = 0.034, Mann–Whitney-Test) (Fig. 2D), pointing towards peripheral neuropathy rather than isolated radiculopathy causing sensory deficits in patients with higher NIS. Accordingly, CMAPs and SNAPs, as well of conduction velocities of all other nerves (tibial, median and ulnar) were reduced in patients with NIS ≥ 20, with a general picture of primarily axonal neuropathy in these patients (Suppl. Table 1).

### Impact of comorbidities on NIS

**Figure 3:**
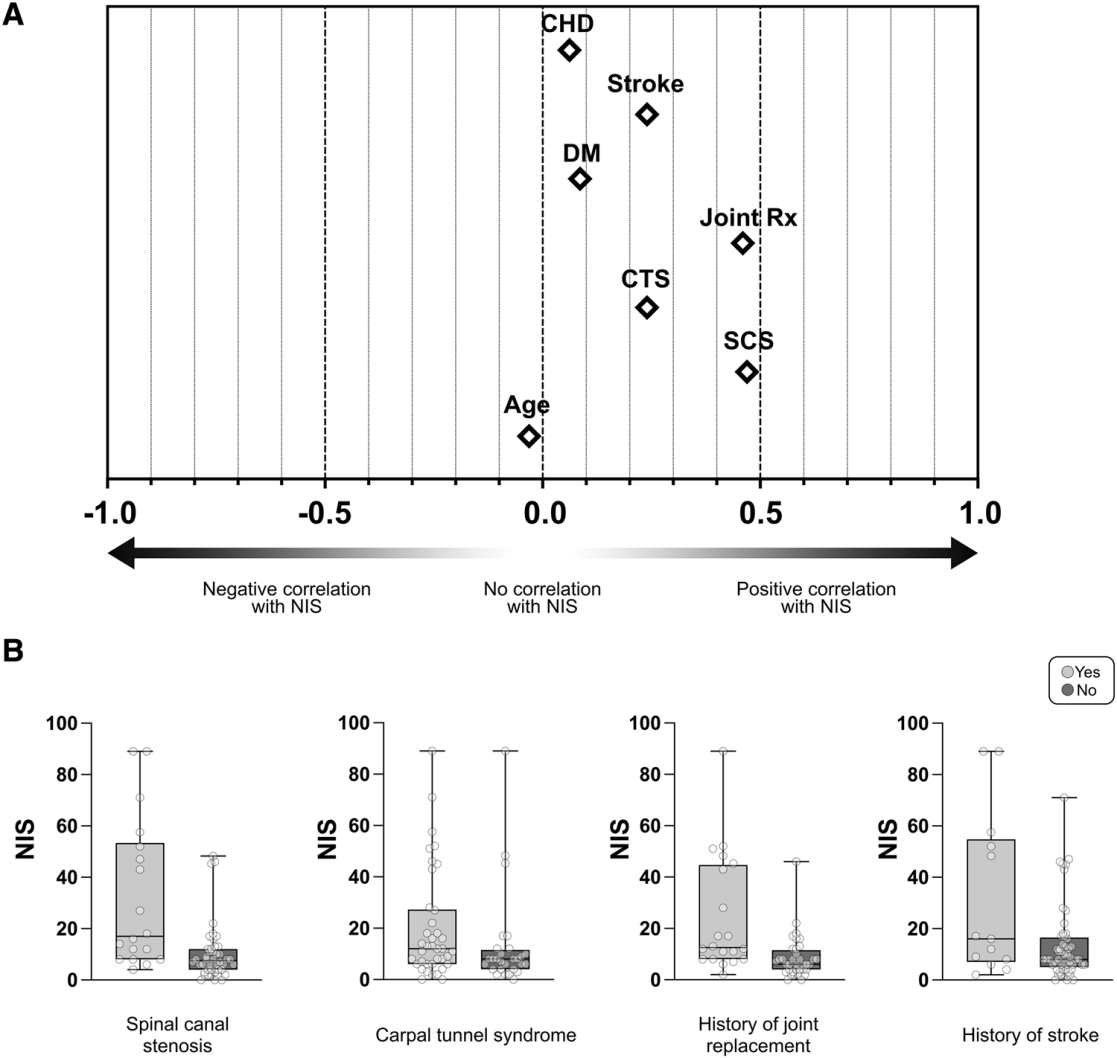
Correlation of comorbidities with NIS. (**A**) Spearman correlation of different comorbidities with NIS. Values of Spearman’s r range from − 1.0 to + 1.0. Diamond shapes indicate the respective correlation coefficients with the NIS. coronary heart disease = CHD; history of stroke = stroke; diabetes mellitus = DM; history of joint replacement = Joint Rx; carpal tunnel syndrome = CTS; spinal canal stenosis = SCS. (**B**) Boxplots depicting NIS scores of patients with or without selected comorbidities. Statistics: Mann–Whitney-U test. Spinal canal stenosis: p = 0.0002; yes: n = 18; no: n = 44. Carpal tunnel syndrome: p = 0.057; yes: n = 38; no: n = 28. History of joint replacement: p = 0.0004; yes: n = 20; no: n = 36.

The average age of patients with A-ATTRwt is high, which suggests an important impact of comorbidities on neurological examination scores such as the NIS. Furthermore, spinal canal stenosis (SCS)^15^, and carpal tunnel syndrome (CTS)^16,17^ are frequent in A-ATTRwt. In order to better understand the relation of comorbidities such as SCS and CTS, but also history of joint replacements, diabetes mellitus (DM), and cardiovascular comorbidities such as history of stroke and coronary heart disease (CHD) with the NIS, we calculated the Spearman correlation coefficient for each of these selected comorbidities with the NIS (Fig. 3A). The highest correlation was seen for SCS (r = 0.47) and history of joint replacement (r = 0.46), although both were only moderate, while CTS and history of stroke showed correlation coefficients of only r = 0.24. Coronary heart disease and DM as well as age did not correlate with NIS (r < 0.1). However, since median HbA1c in the entire cohort was over 5.7% (see Table 1), we presumed a relevant impact of prediabetes in the cohort. Indeed, out of 45 patients for whom HbA1c values were available, only 14 (31.1%) showed an HbA1c lower than 5.7%. However, similar to diagnosed DM, HbA1c did not correlate with NIS (r = 0.12). Furthermore, patients with HbA1c under 5.7% and patients with HbA1c equal to or above 5.7% showed similar median NIS sum scores (median NIS in patients with HbA1c < 5.7% = 12.5, IQR 8.5, n = 14; median NIS in patients with HbA1c ≥ 5.7% = 12.0, IQR 21.5, n = 31). To further investigate if NIS sum scores are influenced by SCS, CTS, history of joint replacement, and history of stroke, we compared the average NIS of patients with each comorbidity to patients who had no known diagnosis of that specific comorbidity (Fig. 3B). As expected, only patients with SCS and history of joint replacement showed relevantly higher NIS compared to patients without the comorbidities.

### Serum neurofilament light chain

**Figure 4:**
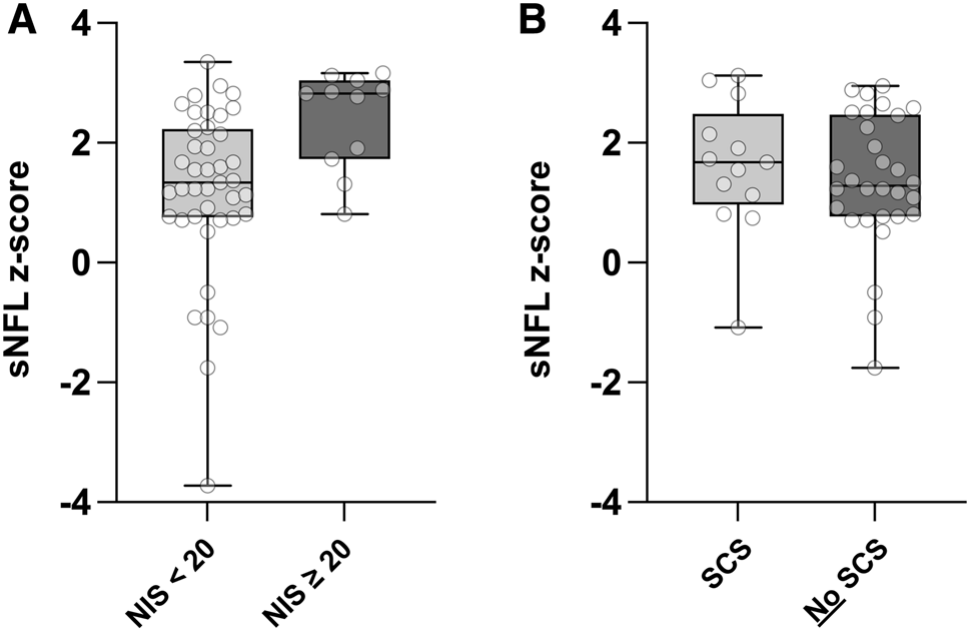
Comparison of serum NFL levels in patients with A-ATTRwt with different severity of neuropathy. (**A**) Boxplots comparing serum neurofilament light chain (sNFL) age and BMI adapted levels (z-scores) as described by Benkert et al.^19^ of patients with NIS < 20 and NIS ≥ 20. Statistics: unpaired t-test; p = 0.010; NIS < 20: n = 41; NIS ≥ 20: n = 11. (**B**) Boxplots comparing sNFL z-scores of patients with spinal canal stenosis (SCS) and without SCS. Statistics: unpaired t-test; p = 0.526; SCS: n = 13, no SCS: n = 30.

Neurofilament light chain has been described as a biomarker in several types of peripheral neuropathies including inherited transthyretin amyloidosis (A-ATTRv)^12,13^. We therefore analyzed the serum NFL levels of patients with severe neuropathy in comparison to our control cohort (Fig. 4A). Since NFL is secreted by neurons constantly in low levels in an age-dependent manner^18^ and has been described to negatively correlate with body mass index (BMI), we calculated individual z-scores to standardize the NFL levels of our cohort using the reference data and online application published by Benkert et al.^19^. Age- and BMI-adapted serum NFL levels were compared in patients with NIS < 20 and patients with NIS ≥ 20 (Fig. 4A). In Patients with NIS ≥ 20, z-scores of serum NFL were notably increased in comparison to patients with NIS < 20 (NIS < 20: mean 1.2, SD 1.4, n = 41; NIS ≥ 20: mean 2.4, SD 0.8, n = 11; p = 0.010, unpaired t-test, Fig. 4A). Since not only polyneuropathy but also other (central) neurological disorders can cause elevation of NFL^20^, and especially SCS has shown to have a relevant effect on the NIS, we evaluated if patients with SCS have higher levels of age- and BMI-adapted serum NFL compared to patients without SCS. There is no difference in z-scores of serum NFL between patients with and without known SCS (SCS: mean 1.6, SD 1.1, n = 13, No SCS: mean 1.4, SD 1.1, n = 30; p = 0.526, unpaired t-test, Fig. 4B).

## Discussion

Peripheral neuropathy is an accepted aspect of A-ATTRwt^2–6^. However, only single cases of severe neuropathy have been reported to date^8–11^. In this prospective study we evaluated a cohort of patients with severe neurological affection in A-ATTRwt in order to better understand the phenotypes and etiologies underlying high neuropathy scores compared to patients with mild scores by analyzing a set of comprehensive clinical and patient-reported outcomes. 73 patients out of the entire cohort of 134 patients that were recruited to the ACCB Registry between 2018 and 2023 had confirmed A-ATTRwt and complete NIS datasets at the time of study evaluation. In the representative cohort of patients included, 16/73 (21.9%) presented a NIS of 20 points or more. Although peripheral neuropathy, especially idiopathic axonal neuropathy, is also frequent in the general population of the elderly (up to 20% under the age of 80 and up to 40% over the age of 80)^21^, the degree of disability represented by a NIS of 20 or more points clearly exceeds that of idiopathic neuropathy of the elderly. Importantly, this finding resonates with previous studies, that have shown a comparably high prevalence of peripheral neuropathy in A-ATTRwt patients in about 30%^7^ to 50%, which led to change of clinical management in almost half of the patients^5^. A study of Campagnolo et al. reported NIS scores of 20 or higher in almost 20% of patients (3 of 16), similar to our results^4^.

In our study, 90% of patients were elderly male, which is typical for A-ATTRwt^22^. Although there was no drastic difference, patients with more severe neuropathy tended to be slightly younger at time of diagnosis than patients with mild neuropathy symptoms, while both groups had similar percentages of women. Even though the data revealed a very slightly negative correlation of age and NIS, there is likely no noteworthy correlation. Interestingly, previous studies have suggested that age of onset of neuropathy may precede cardiomyopathy^7^. Although we did not find a difference in diagnostic delay between patients with mild and severe neurological symptoms in our study, it is important to consider neurological aspects for the diagnosis of A-ATTRwt. This is underlined by the fact that patients with NIS ≥ 20 scored markedly worse in quality-of-life-assessments (RODS, Norfolk). Although not statistically different, higher median NTproBNP levels in patients that are also neurologically more affected suggests a systemic rather than only cardiac disease that is generally more advanced in those patients and has important implications for risk of mortality^23^. In order to better understand the origin of worse NIS, we compared the clinical phenotype as well as comorbidities between patients with NIS < 20 and patients with NIS ≥ 20.

Clinical characterization of patients with “typically” mild affection (NIS < 20) reproduces the previously described classical neuropathy phenotype of mild sensory ataxia^2^. The clinical picture of patients with NIS ≥ 20 in our cohort depicts a length-dependent, axonal neuropathy with distally pronounced paresis, sensory deficits, and barely any affection of cranial nerves, which resembles the peripheral neuropathy classically seen in late-onset A-ATTRv patients^24^. However, the NIS is a score that includes, among other modalities, the subjective quantification of motor strength and reflex elicitation, which is highly dependent on the abilities and strength of the examiner^25^. This renders it difficult to derive conclusions about the cause of the observed deficits. Furthermore, the neuropathy score is prone to confounding by other causes of weakness, and results are dependent on the patient’s cooperation, which may not always be sufficient in a cohort of multimorbid patients with an average age around 80 years. Due to the high average age, patients with A-ATTRwt are prone to comorbidities affecting clinical neurological scores^3–6,15,17,26,27^. In fact, our study showed that high NIS correlated with SCS, which can cause radiculopathy, and history of joint replacement that influences strength and mobility. SCS is an important aspect of A-ATTRwt^15,17,27^, and has been described to have a prevalence of just over 10%^2,4^ to 50%^7^ in patients with A-ATTR in recently published studies. Joint replacement has been reported to be more common in patients with A-ATTR-cardiomyopathy and to even precede cardiac symptom onset^28^. Others have found amyloid deposits in synovial membranes that were surgically removed during arthroplasty^29–31^. The higher incidence of arthroplasty may therefore be associated with systemic amyloidosis in our cohort, but histological proof would be necessary to support this assumption. Diabetes mellitus (DM) is another etiology that is likely to contribute to neuropathies and high NIS. In our study, however, DM did not correlate with NIS by itself and HbA1c levels did not differ relevantly between groups of NIS < 20 and NIS ≥ 20. While in our cohort, DM does not correlate with the NIS, diabetic neuropathy may still be a factor and complication of neuropathy. Blood sugar management should therefore be an important aspect of follow-up in A-ATTRwt.

Despite the importance of comorbidities for neurological symptoms in A-ATTRwt, it could be speculated that amyloidosis, as a systemic disease, contributes to neurological symptoms by damaging peripheral nerves. Interestingly, while SCS can be a potential cause for the significantly worse peroneal amplitudes in more severely affected A-ATTRwt patients, sural nerve amplitudes were also markedly reduced in patients with NIS ≥ 20. This suggests a role of the peripheral neuropathy rather than an isolated polyradiculopathy causing the clinical symptoms. Could amyloid depositions therefore contribute to the neuropathy in the higher affected subgroup of patients with A-ATTRwt? Up to now, amyloid depositions on peripheral nerves can only be proven by peripheral nerve biopsies, while both endomysial and perivascular deposits of TTR amyloid can occur^32^. However, nerve biopsies are invasive procedures leading to permanent loss of local innervation, and the patchy distribution of amyloid deposition may lead to false negative results^33–35^. Because of the small diagnostic yield and invasiveness of the procedure, patients in this study did not undergo systematic peripheral nerve biopsies. For reliable diagnosis of TTR neuropathy, easily accessible, non-invasive, and sensitive biomarkers for amyloid neuropathy are essential. Soft-tissue uptake in DPD scintigraphy has been suggested to correlate with myopathy^9^ and neuropathy in A-ATTR^36^, although localization to single peripheral nerves is difficult and would require SPECT/CT imaging, which is seldom performed in the lower extremity^37^. Therefore, although some results of our study may suggest it, a causal association of amyloidosis and neuropathy remains uncertain. However, biomarkers indicating progress and therapy response of ATTR neuropathy have been of paramount interest in A-ATTRv and other neuropathies, especially serum NFL^12–14^. Serum NFL has also shown response to treatment with A-ATTR-specific therapies^14^. Our study shows that neurological symptoms in A-ATTRwt are a relevant aspect of the disease and significantly affect quality of life, illustrating the need for a biomarker of neurological progression and response of neurological symptoms. We show that serum NFL is a promising biomarker for clinical neurological affection in A-ATTRwt, as patients with NIS ≥ 20 points have higher age- and BMI-adapted serum NFL levels (z-scores) than patients with NIS < 20^19^. Interestingly, the presence or absence of SCS alone, which may cause increased NFL through spinal cord compression, did not cause a difference of serum NFL levels, which suggests that neuropathy is the source of elevated levels in patients with higher neuropathy scores. Importantly, a recent study by Capo et al. illuminated not only the increase of serum NFL levels with age, especially in men, but also negative correlations of serum NFL levels to muscle mass and strength^38^. Our results go in line with this study and strengthen the claim of serum NFL as a marker for neurodegeneration. However, while adaptation of serum NFL levels to age and BMI is important, normative values for z-score calculations for age and BMI adaptation of serum NFL values were generated with younger patients in general^19^. Therefore, future reference data including older patients will be necessary to validate our findings. Additionally, it has to be taken into account that renal function can affect serum NFL levels^39^, which was not assessed in our study. Although the necessity of adjusting to renal function has been considered unnecessary when comparing serum NFL levels to other neurodegeneration markers, as we considered the NIS in this study^39^, renal function may still have an impact on serum NFL levels, and should be considered in future studies.

Our exploratory study had limitations. As we were performing a monocentric study, patient numbers were small, which restricts the generalizability of our results. Furthermore, some data were unavailable for single patients of our cohort. Potential reasons for missing data are mainly limited compliance completing questionnaires and increased effort of traveling to the study center, especially for severely affected patients. The large percentage of patients not included into the study (61/134 patients) was due to lack of informed consent, missing NIS-score, or not (yet) confirmed diagnosis of A-ATTRwt. This underlines the difficulty to recruit large cohorts in rare disease, especially in an elderly and diseased population that frequently suffers from comorbidities. With a larger number of patients, extended regression models could have been calculated accounting for influential variables on the respective outcomes. Nevertheless, considering A-ATTRwt is a rare disease, we were able to provide valuable new insights and demonstrate a relevant impact of neurological symptoms and especially neuropathy in patients with A-ATTRwt.

In conclusion, symptoms of severe neuropathy are relevant and underestimated features in A-ATTRwt. While the NIS is a valuable tool for evaluating the severity of patients’ symptoms, it also reflects comorbidities such as spinal canal stenosis and past orthopedic surgery, and therefore must be regarded with care when scoring peripheral neuropathy in A-ATTRwt patients. Nevertheless, our study suggests that serum NFL is a promising biomarker for peripheral neuropathy in A-ATTRwt. Further studies will be necessary to understand the pathogenesis of neuropathy in A-ATTRwt and to develop more specific biomarkers and therapeutic options for patients with severe neuropathy in A-ATTRwt.

## Methods

### Patients

Patients were prospectively recruited between 2018 and 2023 in context of the Amyloidosis Registry Study at Charité Berlin (Campus Benjamin Franklin, Campus Charité Mitte, and Campus Virchow Klinikum). The study was approved by the local ethics committee and informed consent was given. Out of a total of 134 patients was recruited into the registry databank, for 73 patients informed consent was obtained to participate in the study, diagnosis of A-ATTRwt was confirmed, and a complete NIS necessary to assign patients to a group (NIS < 20; n = 57 or NIS ≥ 20; n = 16) was available. According to the general consensus^1,40^, diagnosis of A-ATTRwt was established by proof of TTR in tissue biopsy and/or tracer uptake in DPD-Scintigraphy plus negative monoclonal protein detection tests, along with negative genetic testing for *TTR* mutations. Genetic testing was performed using Next Generation Sequencing (Centogene GmbH, Rostock, Germany) as described before (see Kleefeld et al.^2^). Patients with a NIS of less than 20 points were classified as mildly affected, while patients with a NIS of 20 points and higher were labelled as severely affected. Disease onset was determined by patient history (begin of subjective deterioration of cardiac function leading to diagnosis), time of diagnosis was defined by the date of consensus and therapy start determined by ACCB board after evaluation of all diagnostic criteria.

### Neurological examination and scores

Standardized neurological examination was performed in all patients. The examination included exploration of subjective symptoms, examination of basic mnestic abilities and communication, cranial nerves, sensory and motor testing, as well as coordination and gait. For scaling peripheral neuropathy, we used NIS^25^, which is the general standard score used to score neuropathy in A-ATTR, including scoring in clinical trials for ATTRv neuropathy^2,41,42^. The NIS is composed of scoring motor and sensory exams as well as reflexes resulting in a maximum score of 244 points. NIS was conducted by the same assessor for consistency.

### Electrophysiological examination

Electrophysiological examination was performed using a standardized protocol for nerve conduction studies (NCS) of medial, ulnar, tibial, peroneal, and sural nerves as described before^2^.

### Quality of life assessment

Validated questionnaires were used for assessment of life quality and ability to perform daily activities. For quality of life assessment, Norfolk Quality of Life (Norfolk QoL)^2,43–45^ and the Rasch-Built-Overall-Disability Score (RODS)^43,46^ were used.

### Laboratory examination

Laboratory examinations involved blood and urine analysis. We measured standardized cardiac markers including troponin t, N-terminal prohormone of brain natriuretic peptide (NTproBNP), as well as vitamin B12, and glycated hemoglobin (HbA1c). Furthermore, we screened for free light chains in serum and immunofixation electrophoresis to exclude paraproteinemia. Serum neurofilament light chain (sNFL) levels were measured using Single molecule array (Simoa). Age- and BMI-adapted sNFL z-scores were calculated using the sNFL Reference App online service (https://shiny.dkfbasel.ch/baselnflreference/) as described by Benkert et al.^19^.

### Cardiac assessment

For cardiac assessment, all patients underwent clinical examinations. Tissue biopsies were taken within the regular diagnostic workup. Endomyocardial biopsies were taken of the left ventricle as described before^2^.

### ^99m^Tc-DPD scintigraphy

Technetium-99m-labelled 3,3-diphosphono-1,2-propanodicarboxylic acid (DPD) scintigraphy and hybrid single-photon emission computed tomography (SPECT/CT) were performed and evaluated using standardized protocols as previously described^47^. Heart to contralateral (H/CL) ratios were calculated after quantification as described before^47^.

### Statistics

Variables were described descriptively by providing mean and standard deviation, median and interquartile range or absolute and relative frequencies respectively. Characteristics are also reported separately for the two subgroups of NIS below a score value of 20 and at least a score value of 20.

The sample size (n = 73) of this exploratory study was based on all patients of the Amyloidosis Registry Study at Charité Berlin (Campus Benjamin Franklin, Campus Charité Mitte, and Campus Virchow Klinikum) recruited between 2018 until 2023 where informed consent was obtained and a diagnosis of A-ATTRwt was confirmed as well as they were attributable to one of the two groups (i.e., available NIS score). Due to the exploratory character of the study, statistical test results are only to be interpreted in the respective light and no adjustment for multiple testing was conducted. Student’s t-test or the Mann–Whitney-U test (chosen based on scale level, and data distribution evaluated descriptively by histogram, measures of location and skewness) were applied to the research questions for comparing the two groups of patients with mild neurological affection (NIS < 20) and patients with significant neurological affection (NIS ≥ 20). Due to the very limited sample size (n = 16 with a NIS values of at least 20) no regression models were applied with adjustment for potential influential variables. Nevertheless, Spearman’s correlation coefficients were calculated in order to get first ideas regarding potential relationships between NIS and comorbidities as well as demographics. Potential missing values are reported for each analysis. Missing values were not imputed.

Statistical analysis and graphs were created by using GraphPad Prism (Version 10.0, GraphPad, San Diego, CA, USA) and R 4.2.2 via RStudio (Version 2023.09.1 + 494, Posit PBC, Boston, MA, USA). Figure labels, legends and arrangements were done using Pixelmator Pro (Version 3.4 Camelot, Pixelmator Team Ltd, Vilnius, Lithuania).

### Ethics approval

This study followed ethical principles of the Declaration of Helsinki ratified by the World Medical Association in 1964. The study was approved by the local ethical research committee, Ethical committee of Charité – Universitätsmedizin Berlin (reference number: EA1/014/20).

### Supplementary Information

Supplementary Table 1.

## Data Availability

The data that support the findings of this study are not openly available due to reasons of sensitivity and are available from the corresponding author upon reasonable request. Data are located in controlled access data storage at Charité—Universitätsmedizin Berlin. Data can be requested by contacting Dr. Katrin Hahn via  katrin.hahn@charite.de .

## Abbreviations

ACCB: Amyloidosis Center Charité Berlin
A-ATTRwt: Wild type transthyretin amyloidosis
A-ATTRv: Hereditary transthyretin amyloidosis
NIS: Neuropathy Impairment Score
NFL: Neurofilament light chain
