# Supplementary material for: Neurological affection and serum neurofilament light chain in wild type transthyretin amyloidosis
Source: Sci Rep. 2024 May 2;14:10111. doi: 10.1038/s41598-024-60025-6 (PMC11066119; doi:10.1038/s41598-024-60025-6)
Supplement: Supplementary file 1 — Supplementary Table 1. [file 41598_2024_60025_MOESM1_ESM.pdf]

Supplementary Information

Suppl. Table 1: Nerve conduction studies of median, ulnar, and tibial nerves. IQR: interquartile range. P-values were calculated using Mann-Whitney-U test. CMAP: muscle action potential; SNAP: sensory nerve action potential; CV: conduction velocity; dML: distal motor latency.

Nerve	NIS < 20			NIS ≥ 20			p-value
	Median	IQR	n	Median	IQR	n	
	CMAP						
Tibial	3.6	2.7	22	1.3	2.5	7	0.013
Median	5.0	1.6	26	4.9	5.5	10	0.645
Ulnar	7.8	2.0	24	7.1	2.2	10	0.064
	dML						
Tibial	4.6	1.5	19	7.5	4.3	7	0.104
Median	4.1	0.9	19	5.2	2.6	8	0.354
Ulnar	2.9	0.6	17	3.0	0.8	8	0.367
	Motor CV						
Tibial	41.7	8.0	24	31.6	10.8	10	0.003
Median	50.6	12.7	26	44.8	7.2	10	0.174
Ulnar	52.8	8.8	24	49.7	13.5	10	0.206
	SNAP						
Median	6.6	4.5	15	5.3	9.1	7	0.744
Ulnar	16.3	14.4	7	6.7	10.2	6	0.160
	Sensory CV						
Median	49.1	18.0	15	47.2	24,7	7	0.298
Ulnar	55.9	13.4	14	30.9	38.1	5	0.007
